# Co-creation and priority setting for applied and implementation research in One Health: Improving capacities in public and animal health systems in Kenya

**DOI:** 10.1016/j.onehlt.2022.100460

**Published:** 2022-11-17

**Authors:** Folorunso O. Fasina, Mark Nanyingi, Rinah S. Wangila, Stephen Gikonyo, Ruth Omani, Thomas Nyariki, Lucy W. Wahome, Joy Kiplamai, Evans Tenge, Fredrick Kivaria, Sam Okuthe, Serge Nzietchueng, Tabitha Kimani, Joshua Kimutai, Gerald Mucheru, Obadiah Njagi, George Njogu, Robert Rono, Grace N. Maina, Dan Mogaka, Joseph Mathooko, Mohammed M. Sirdar, Eddy G.M. Mogoa, Angela Makumi, Bernard Bett, Athman Mwatondo, Victoria Kanana Kimonye, Innocent B. Rwego, Abdirahman Adan, Samuel Wakhusama, Patrick Bastiaensen, Charles Bebay

**Affiliations:** a Emergency Center for Transboundary Animal Diseases (ECTAD), Food and Agriculture Organization of the United Nations (FAO), United Nations Office in Nairobi (UNON), Gigiri, Nairobi, Kenya; b Faculty of Health and Life Sciences, Department of One Health, University of Liverpool, Liverpool, UK; c Emergency Center for Transboundary Animal Diseases (ECTAD), Regional Office for Eastern Africa, Food and Agriculture Organization of the United Nations (FAO), United Nations Office in Nairobi (UNON), Gigiri, Nairobi, Kenya; d Directorate of Veterinary Services (DVS), Ministry of Agriculture, Livestock, Fisheries and Co-operatives, Nairobi, Kenya; e Department of Health Services, Baringo County Government, Kabarnet, Kenya; f Directorate of Veterinary Services, Murang'a County Government, Murang'a, Kenya; g World Health Organization (WHO), World Health Emergencies (WHE), United Nations Office in Nairobi (UNON), Gigiri, Nairobi, Kenya; h Inclusive Value Chain, Food and Agriculture Organization of the United Nations (FAO), United Nations Office in Nairobi (UNON), Gigiri, Nairobi, Kenya; i Sub-Regional Representation for Southern Africa, World Organization for Animal Health, Gaborone, Botswana; j Africa One Health University Network (AFROHUN) Kenya, Faculty of Veterinary Medicine, University of Nairobi, Nairobi, Kenya; k International Livestock Research Institute (ILRI), Nairobi, Kenya; l Zoonotic Disease Unit (ZDU), Ministry of Health, Nairobi, Kenya; m Global Health Security Agenda, Ministry of Health, Afya House, Nairobi, Kenya; n CORE Group Polio-Global Health Security Project, Kenya and Somalia, Nairobi, Kenya; o Sub-Regional Representation for Eastern Africa, World Organization for Animal Health, Nairobi, Kenya

**Keywords:** Anthropology, Community, Co-creation, Decision making, Development agenda, Ecosystem health, Empowerment, Gender-based approach, Global health security, Human right, Inclusive training, Indigenous peoples, Integration, Kenya, One health, Participatory research, Prioritization, Public participation, Sustainable environment

## Abstract

**Background:**

The Kenyan government has successfully been implementing sector specific and multisectoral projects aligned to the Global Health Security Agenda (GHSA). For operational readiness and to enhance the effective planning and implementation of Global Health Security Programs (GHSP) at national and subnational level, there is an urgent need for stakeholders' engagement process to seek input in identifying challenges, prioritise activities for field implementation, and identify applied research and development questions, that should be addressed in the next five years.

**Methods:**

The modified Child Health and Nutrition Research Initiative (CHNRI) method was used to identify global health security related priorities for multisectoral implementation in Kenya. Subject matter experts from human, animal and environmental health sectors at national and subnational level contributed to predefined research questions from a number of sources and activities for consideration for implementation using a One Health approach. Sixty-two experts scored the 193 questions based on five pre-defined criteria: 1) feasibility and answerability; 2) potential for burden reduction; 3) potential for a paradigm shift; 4) potential for translation and implementation; and 5) impact on equity. Data resulting from this process was then analysed in a Microsoft Excel spreadsheet to determine the research priorities and experts' agreements.

**Results:**

Among the priority activities identified for implementation research were; strengthening One Health governance and legal frameworks; integration of ecosystem health into One Health programming; strengthening disease reporting, integrated data collection, information sharing and joint outbreak response; socio-anthropological and gender-based approaches in improving risk and behavioural change communication and community engagement; and one health workforce development. In addition, the potentials to invest in collaborative predictive risk modelling to enhance epidemic intelligence systems, while strengthening the One Health approach in the food safety incident and emergency response plans are feasible.

**Interpretation:**

Successful multisectoral implementation of global health security program in Kenya calls for a whole of society approach that will harness community and private sector knowledge to build preparedness and response capacities while targeting neglected and marginalised populations. This research provides a framework that is worth emulating for cost-effective planning and implementation of overarching One Health programs.

## 1 Introduction

The International Health Regulations (2005) (IHR (2005), a legally binding agreement among 196 countries globally, was set with the purpose to prevent, protect against, control and provide a public health response to the international spread of disease in ways that are commensurate with and restricted to public health risks, and which avoid unnecessary interference with international traffic and trade [1]. Since its adoption, countries have undertaken the Joint External Evaluation (JEE), a voluntary, collaborative, multisectoral process to assess country capacities to prevent, detect and rapidly respond to public health risks. This process serves as a tool to monitor and evaluate gaps found during the JEE and implement recommendations arising from the process [2]. Complementary to the JEE process is the Performance of Veterinary Services (PVS) tool, which is used to evaluate country's veterinary services as contributors to animal and public health capacities [3]. The Global Health Security Agenda (GHSA) is a partnership of more than 70 countries working collaboratively to address global health threats posed by infectious diseases [4]. The JEE assists countries to assess their health security strengths, weaknesses, opportunities and threats while the GHSA assist countries to mobilise and direct resources to leverage on and complements the existing strengths and opportunities, and at the same time address weaknesses, gaps and threats through targeted capacity building and leadership in the prevention and early detection of, and effective response to, infectious disease threats.

As part of efforts to address the health security challenges identified in the Kenya's JEE [5], the country developed the *National Action Plan for Health Security (NAPHS) (2019–2023)*, a country owned, multi-year, planning process that is based on One Health for all-hazards for implementation. Basically, NAPHS is one cardinal means of implementing the GHSA and IHR (2005) recommendations. The country is also implementing the GHS, through funding from United States Agency for International Development (USAID), and multiple partnerships, using a One Health approach. Recently, the One Health High-Level Expert Panel (OHHLEP) redefined One Health as an integrated, unifying approach that aims to sustainably balance and optimize the health of people, animals, and ecosystems. It recognizes the health of humans, domestic and wild animals, plants, and the wider environment (including ecosystems) are inextricably linked and interdependent. The underlying principles for this new definition are disciplinary and sectoral equity, socio-political and multicultural parity, socio-ecological equilibrium, stewardship of responsible human behaviour and sustainability, and trans-disciplinary and multisectoral collaboration [6]. Basically, this new definition applies the whole of society approach in the implementation of projects utilizing the One Health approach. This is significant considering that this approach has been heralded as having benefits that include improved coordination, collaboration and communication in addressing, and more effectively and holistically, shared public health threats at the animal-human-environmental interface [7].

In compliance with the new definition of One Health and with the requirements of IHR (2005), the co-creation, prioritization and alignment of One Health research agenda and implementation in Kenya are important. It is on this basis that the FAO called for a multisectoral-stakeholders' consultation to co-create and set prioritised One Health research and development agenda for Kenya for the next five years, 2023–2027. The end goal is to ensure that the health investments in the country meet the key objectives, are impactful and are contributing to the sustainable development goals (SDGs). Furthermore, the outcome of this novel multidisciplinary approach will establish a replicable empirical framework that will contribute to evidence based decision making and cost-effective one health programming and policy making in human, animal and environmental health sectors at the national and subnational level in resource limited settings.

## 2 Materials and method

We utilised the Child Health and Nutrition Research Initiative (CHNRI) method [8,9], that was originally developed for setting priorities for health research investments aimed to reduced global child mortality. To enhance its applicability to the local scenarios, we modified it to suit a One Health context by categorizing the inputs along One Health thematic areas, with the questions contextualised to the Kenyan scenario. Based on evaluation of the tool, it allows for systematic, transparent, replicable, and has clearly defined context and criteria, which involves the adoption of the whole-of-society approach including funders, stakeholders and policymakers, and is a structured way to obtain reliable information. Furthermore, it is informative and generates intuitive quantitative outputs; it also allows for the studying of the level of agreement over each proposed research idea; and the initial independent scoring by many experts limits the influence of individuals on the rest of the group [8,9].

## 3 Development of the questionnaire

Through extensive consultation and literature reviews, a list of research agenda, relevant activities, One Health challenges, implementation science and applied research questions (*n* = 193) was harmonized. They broadly covered six thematic areas on; i) establishing foundations of One Health capacities ii) reducing risks from emerging and re-emerging zoonotic infectious diseases with pandemic potential, iii) mechanisms for controlling and eliminating endemic zoonoses and neglected tropical diseases (NTDs) and vector borne diseases (VBD), iv) strengthening the assessment, management, and communication of food safety risks, v) curbing the silent pandemic of antimicrobial resistance (AMR), and vi) Integrating the environmental sector into One Health.

The list of questions was synthesised primarily from the following documents: a) the draft One Health Joint Plan of Action 2022–2026 (OH JPA), b) the Kenya PVS report (2019), the Kenya NAPHS (2019–2023), c) the Kenya JEE report (2017), d) Kenya One Health National Strategy (2021–2025), e) the One Health multisectoral One Health coordination mechanisms operation tool (MCM OT) (2022), and f) the implementation agenda put forward previously through consultative process by One Health stakeholders in Kenya [5,[10], [11], [12]]. All questions were placed in the Microsoft Excel spreadsheet and reviewed internally by three FAO subject matter experts in veterinary medicine and epidemiology, One Health, quantitative epidemiology, public and global health, infectious diseases modelling (Supplementary Table 1). Taking into account the geographical and political contexts of Kenya (national and subnational systems) and the critical One Health needs of the society, five criteria and four scores (1.0; 0.5; 0.0 and blank) were established according to previously established guidelines [8,9; Supplementary Table 1]. Briefly, each question was qualitatively evaluated against the five predefined criteria as follows:

a) Feasibility and answerability – “Would you say that the proposed contribution or research area would likely be feasible and successful in reaching the proposed endpoint and delivering public good in Kenya?”
b) Potential for burden reduction – “Would you say that this contribution or research area has the potential to markedly reduce the burden of zoonoses, and AMR, and promote antimicrobial stewardship, multisectoral collaboration, transdisciplinary engagement and One Health in Kenya?”
c) Potential for a paradigm shift – “Would you say that this contribution or research area is likely to result in a ‘paradigm shift’ that could change and improve our current understanding of the problem of zoonoses and AMR, and promote antimicrobial stewardship, multisectoral collaboration, transdisciplinary engagement and One Health in Kenya?”
d) Potential for sustainability, translation and implementation – “To the best of your knowledge and experience, would you say that the proposed contribution or research area would likely lead to practical application, implementation of new knowledge, and/or be deliverable at scale?”
e) Impact on equity – “Would you say that the proposed contribution or research area would likely improve equity in the society with regards to the social strata, economic class, livelihoods, gender, youth inclusion, and possibly adopt a whole of society approach?” [8,9].

Scores were allocated by respondents using the following instructions: i). If you agree that the research question would satisfy the priority setting criterion, please enter “1.0” in the relevant cell; ii). If you disagree, please enter “0.0”; iii). If you are knowledgeable about the question, but can neither agree nor disagree, then please enter “0.5”. However, please try to avoid using “0.5”, because this choice is the least helpful in enabling us to discriminate between the many competing research ideas; iv). If you don't have enough knowledge on the research topic to judge, then please leave the cell blank and move to the next cell [9]. This was consistent with original instructions in the use of the CHNRI tool originally.

The tool was piloted among seven experts (two veterinarians, one each of laboratory scientist, epidemiologist, animal production expert, antimicrobial resistance specialist and an environmental scientist at national and county-levels) to evaluate for consistency and clarity, the average time of completion of the questionnaire, best mode of presentation to the stakeholders and other associated logistics. Thereafter, the *Emergency Center for Transboundary Animal Diseases* (ECTAD) team of four experts (FOF, MN, RSW and SG) conducted exploratory discussions to consider all the feedbacks, utilised them to adjust the tool to make it fit for field application, encourage stakeholders' responses, and build a library of terms (Supplementary Table 2). We adopted a hybrid approach of sharing (distribution in the emails using detailed information) and conducting of a stakeholders' consultation workshop.

## 4 Establishment of a pool of subject matter experts

We established a multidisciplinary pool of experts relevant to Kenya context, including experts living within or outside the country but who have experience on biomedical research or health-related and developmental works in Kenya (*n* = 183). To qualify for inclusion in the pool of experts, each candidate should have fulfilled at least one of the following criteria: a) working for the Government of Kenya at the national or subnational level as a health or biomedical professional; and those in animal production, animal health, crop agriculture, environment, extension services, and such related fields; b) working as development or donor partner in Kenya; c) working with the Community-based Organisations (CBOs), Faith-based Organisations (FBOs) or local or International Non-governmental Organisations (NGOs or INGOs); d) working as private medical or veterinary practitioners with direct interface with the field; e) working with the universities or relevant research institutions or other tertiary colleges; f) have significant peer-reviewed or popular article or publication in the field of zoonoses, environment, global health, One Health, public health, antimicrobial resistance, wildlife research, multidisciplinary or transdisciplinary sciences within the last five years.

Preliminary consultation was conducted through crowdsourcing attempts by email to each of the respondents. To ensure high compliance rate for completion of the questionnaire, a multisectoral stakeholders' consultation was later held in Nairobi for 53 multidisciplinary experts. Each expert was encouraged to independently score the tool completely according to instructions. To remove or reduce biases in order to avoid influence on the ability, or willingness of participants to answer questions precisely or truthfully, and independently without influence, the questions were completely blinded, and discussions/consultation during the process of filling the responses were discouraged; therefore each respondent only relates with his or her individual responses until when all responses were cumulated together. This could hardly influence the outcome of the process until the final consensus outputs were shared in the plenary discussion.

### 4.1 Data analysis

A total of 60 complete responses (21 by emails and 39 through the workshop) were obtained and based on the inputs from these experts, data were entered into a preformatted analytic Microsoft Excel template developed for this purpose. Some of the experts attending the workshop had previously submitted responses by email and only attended the workshop to be part of the review team for the outcomes of the evaluation. Though we have a pool of 183 experts, some represented the same institution and consultatively submitted only one response per their institution. The “Criterion-Specific Scores” (CSS) were generated by calculating the mean of the individual scores for each research question received from all experts. All CSS ranged from 0.0 to 1.0 (100%). Using the simple composite mean of all five CSS per question, the overall “Research Priority Score” (RPS) was generated for each research question. The “Average Experts' Agreement” (AEA), which was an indicator of the average proportion of scorers that returned the modal (most common) answer for a research question, was also calculated for each research question. This provided an understanding of the level of agreement among scorers and was expressed as the frequency of the mode divided by the total number of scores for the question, as per the following formula:

$$\mathrm{AEA}=\frac{1}{5}\times \sum_{\;q=1}^{\;5}=\frac{N\left(\mathrm{Modal score}\;\mathrm{per}\;\mathrm{question}\right)}{N\;\left(\mathrm{Scorers}\right)}$$

Where ‘5’represents the five criteria, and ‘N' is the total number of experts who provided scores per each question [8,9].

## 5 Results

It took a mean time of 204 ± 56 min to complete the questionnaire (Range: 150 min – 330 min). The respondents were multidisciplinary in nature including but not limited to human medicine, veterinary medicine, social and medical anthropology, environmental science, bioinformatics, animal production, and molecular biology. They had specialised at least in bioinformatics, infectious diseases epidemiology, antimicrobial resistance, mathematical modelling, global and public health, genetics, wildlife medicine, project management, monitoring and evaluation, and One Health. They were drawn from the national and county governments, the private sector, funding agencies, implementing partners and universities.

Using the RPS, the priority One Health research areas, which had an RPS score of 0.96 each, included the following: 1) develop mechanisms to support an overarching One Health governance and legal framework; 2) develop and implement mechanisms and partnerships to review and ensure the integration of ecosystem health and the environment into One Health policies and programmes and ensure equity among sectors and groups in One Health platforms at all levels; 3) support the country to strengthen disease reporting and integrated data collection, information sharing and outbreak response; 4) conduct anthropological and participatory research to identify key risky behaviors, acceptance, and feasibility of risk mitigation measures, and appropriate alternatives, including gender-based approaches and Indigenous Peoples' knowledge; 5) convene relevant sectors to facilitate integrated land (and sea) use planning that incorporates human, animal and environmental co-benefits and yields sustainable land and water management; 6) develop and ensure the inclusion of training for in-service medical, public health and veterinary professionals on the importance of and interlinkages between biodiversity conservation, links between health and the environment, how environmental destruction contributes to disease emergence, and the importance of integrating the environment sector in One Health collaborations; 7) support the development of core modules on environment, biodiversity and ecosystem health in the medical, veterinary and public health academic curricula and research agendas; and 8) identify One Health research gaps and priorities, develop a research agenda and advocate for funding to find sustainable solutions to reduce the risk of disease emergence (Table 1).

**Table 1:**
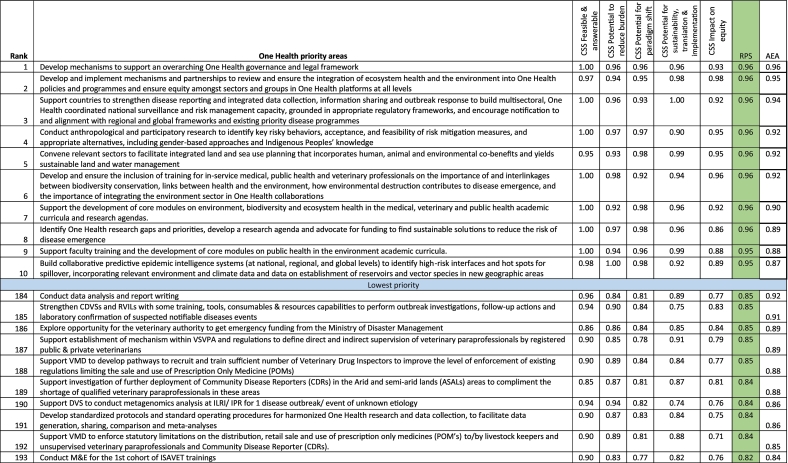
Ranking of One Health research priorities based on Research Priority Scores (partial view – top 10 and bottom 10).

Note that the complete table is available as Supplementary Table 3. CSS = Criterion-specific score; RPS = Research Priority Score; CDVSs = County Directorate of Veterinary Services; VSVPA = Veterinary Surgeons and Veterinary Paraprofessional Act; ISAVET = In-service applied Veterinary Epidemiology Training; VMD = Veterinary Medicine Directorate; M&E = Monitoring and evaluation; DVS = Directorate of Veterinary Services; ILRI = International Livestock Research Institute; RVILs = Regional Veterinary Investigation Laboratories; IPR = Institute of Primate Research. Average experts' agreement scores (AEA) for the top ten agenda ranged between 0.87 and 0.96, and for the bottom ten, the values ranged between 0.84 and 0.92. The table focus is on the green column.

A total of 10 One Health research agenda tied on feasibility and answerability (CSS of 1.00). These were 1) Develop mechanisms to support an overarching One Health governance and legal framework; 2) Support countries to strengthen disease reporting and integrated data collection, information sharing and outbreak response to build multisectoral, One Health coordinated national surveillance and risk management capacity, grounded in appropriate regulatory frameworks, and encourage notification to and alignment with regional and global frameworks and existing priority disease programmes; 3) Conduct anthropological and participatory research to identify key risky behaviors, acceptance, and feasibility of risk mitigation measures, and appropriate alternatives, including gender-based approaches and Indigenous Peoples' knowledge; 4) Develop and ensure the inclusion of training for in-service medical, public health and veterinary professionals on the importance of and interlinkages between biodiversity conservation, links between health and the environment, how environmental destruction contributes to disease emergence, and the importance of integrating the environment sector in One Health collaborations; 5) Support the development of core modules on environment, biodiversity and ecosystem health in the medical, veterinary and public health academic curricula and research agendas; 6) Identify One Health research gaps and priorities, develop a research agenda and advocate for funding to find sustainable solutions to reduce the risk of disease emergence; 7) Support faculty training and the development of core modules on public health in the environment academic curricula; 8) Support improvement of real-time 4-way linkage and information management in veterinary and public health laboratories and epidemiology units to enhance early detection, identification, response and information flow within the system; 9) Support balanced, functional, well-represented national inter-agency coordination mechanisms, and One Health approaches to AMR National Action Plan (NAP) implementation; and 10) Support countries to conduct joint One Health Risk Assessments and mapping leading to evidence-based and targeted risk management and communication (Table 2).

**Table 2:**
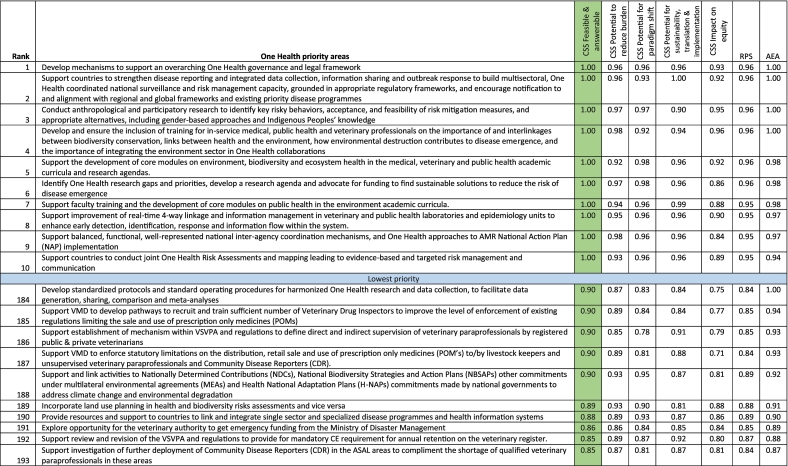
Ranking of One Health research priorities based on Feasibility and answerability Scores (partial view – top 10 and bottom 10).

Note that the complete table is available as Supplementary Table 4. CSS = Criterion-specific score; RPS = Research Priority Score; AMR = Antimicrobial resistance; ASAL = Arid and semi-arid lands; VSVPA = Veterinary Surgeons and Veterinary Paraprofessional Act. Average experts' agreement scores (AEA) for the top ten agenda ranged between 0.94 and 1.00, and for the bottom ten, the values ranged between 0.87 and 1.00. The table focus is on the green column.

Seven research agenda tied in the top position for potentials to reduce burden of infection/mitigate AMR (CSS of 1.00). These seven included the following: 1) build collaborative predictive epidemic intelligence systems (at national, regional, and global levels) to identify high-risk interfaces and hot spots for spillover, incorporating relevant environment and climate data and data on establishment of reservoirs and vector species in new geographic areas; 2) identify actionable solutions/innovative procedures to address the limitation to adopt recommended IPC, including proper vaccination, biosecurity, and food handling practices for identified diseases; 3) provide integrated guidance and resources to countries to help build capacity, empower communities and increase engagement and awareness of endemic zoonotic, neglected tropical and vector-borne diseases prevention, diagnosis, control and treatment; 4) provide technical support and develop training programmes to ensure all countries can conduct food safety risk analysis under a One Health approach; 5) support countries to include or strengthen the One Health approach in the food safety incident and emergency response plans; 6) leverage innovations and new technologies in disease surveillance, rapid response, and control; and 7) engage with local communities including indigenous people, to identify sustainable solutions, nature-based where applicable, for the prevention and control of emerging and re-emerging zoonotic diseases (Table 3).

**Table 3:**
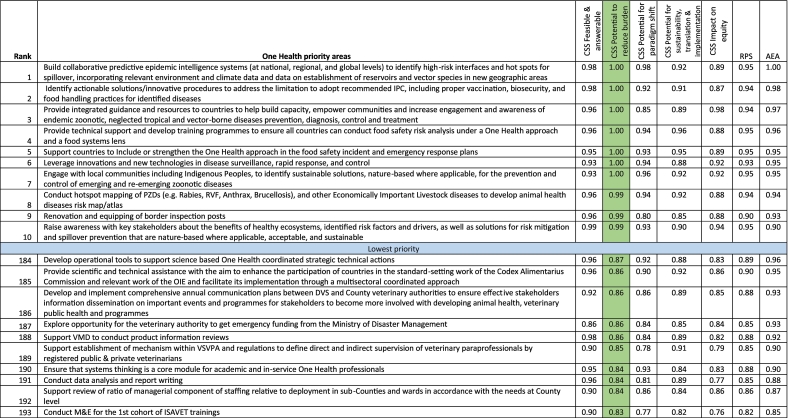
Ranking of One Health research priorities based on potential to reduce the burden Scores (partial view – top 10 and bottom 10).

Note that the complete table is available as Supplementary Table 5. CSS = Criterion-specific score; RPS = Research Priority Score; IPC = Infection prevention and control; PZDs = Prioritised zoonotic diseases; RVF = Rift Valley fever; VMD = Veterinary Medicine Directorate. Average experts' agreement scores (AEA) for the top ten agenda ranged between 0.90 and 1.00, and for the bottom ten, the values ranged between 0.85 and 0.96. The table focus is on the green column.

The top six agenda with potential to cause paradigm shift included the following: 1) map interoperability between health, animal disease and environment databases and information systems (CSS = 1.00); 2) develop joint information management systems and analytical tools integrating ecosystem, environmental, animal and human health knowledge and data (CSS = 0.99); 3) identify One Health research gaps and priorities, develop a research agenda and advocate for funding to find sustainable solutions to reduce the risk of disease emergence (CSS = 0.98); 4) support the development of core modules on environment, biodiversity and ecosystem health in the medical, veterinary and public health academic curricula and research agendas (CSS = 0.98); 5) build collaborative predictive epidemic intelligence systems (at national, regional, and global levels) to identify high-risk interfaces and hot spots for spillover, incorporating relevant environment and climate data and data on establishment of reservoirs and vector species in new geographic areas (CSS = 0.98); and 6) convene relevant sectors to facilitate integrated land and sea use planning that incorporates human, animal and environmental co-benefits and yields sustainable land and water management (CSS = 0.98) (Table 4).

**Table 4:**
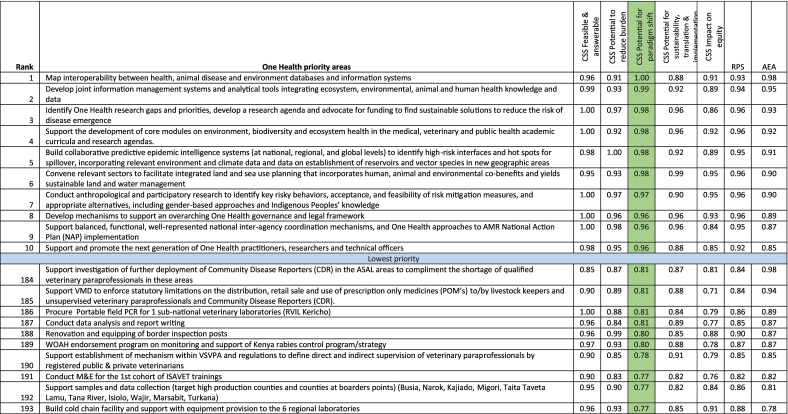
Ranking of One Health research priorities based on potential for paradigm shift Scores (partial view – top 10 and bottom 10).

Note that the complete table is available as Supplementary Table 6. CSS = Criterion-specific score; RPS = Research Priority Score; NAP = National Action Plan on Antimicrobial resistance; CDR = Community Disease Reporters; VSVPA = Veterinary Surgeons and Veterinary Paraprofessional Act; ISAVET = In-service applied Veterinary Epidemiology Training; VMD = Veterinary Medicine Directorate; M&E = Monitoring and evaluation; RVILs = Regional Veterinary Investigation Laboratories; WOAH = World Organization for Animal Health; PCR = Polymerase chain reaction. Average experts' agreement scores (AEA) for the top ten agenda ranged between 0.85 and 0.98, and for the bottom ten, the values ranged between 0.78 and 0.98. The table focus is on the green column.

The emerging priorities (leading five) in regard to potential for sustainability, translation and implementation of any One Health research agenda in Kenya were: 1) supporting the country to strengthen disease reporting and integrated data collection, information sharing and outbreak response to build multisectoral, One Health coordinated national surveillance and risk management capacity, grounded in appropriate regulatory frameworks, and encouraging notification to and alignment with regional and global frameworks and existing priority disease programmes; 2) support faculty training and the development of core modules on public health in the environment academic curricula; 3) convene relevant sectors to facilitate integrated land and sea use planning that incorporates human, animal and environmental co-benefits and yields sustainable land and water management; 4) promote One Health task forces and working groups with clear mandate for internal coordination; and 5) support harmonization of veterinary policies & legislation with other existing laws and regulations (Table 5).

**Table 5:**
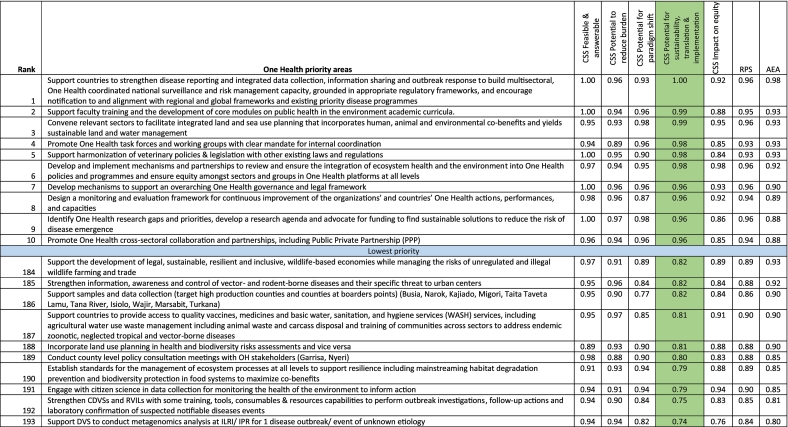
Ranking of One Health research priorities based on potential for sustainability, translation and implementation Scores (partial view – top 10 and bottom 10).

Note that the complete table is available as Supplementary Table 7. CSS = Criterion-specific score; RPS = Research Priority Score; PPP = Public-Private partnership; WASH = Water, sanitation and hygiene services; ILRI = International Livestock Research Institute; IPR = Institute of Primate Research. Average experts' agreement scores (AEA) for the top ten agenda ranged between 0.88 and 0.98, and for the bottom ten, the values ranged between 0.80 and 0.93. The table focus is on the green column.

Of particular interest is the issues that impact on equity, a recently identified necessity in One Health approach based on the definition by OHHLEP [6]. Specifically, the issues raised include: 1) develop frameworks and mechanisms for public participation, including Indigenous Peoples, and horizontal and vertical integration in One Health; 2) provide integrated guidance and resources to countries to help build capacity, empower communities and increase engagement and awareness of endemic zoonotic, neglected tropical and vector-borne diseases prevention, diagnosis, control and treatment; 3) develop appropriate mechanisms/guidelines to ensure participation of indigenous and local communities including their traditional knowledge to guide One Health decision making; 4) develop and implement mechanisms and partnerships to review and ensure the integration of ecosystem health and the environment into One Health policies and programmes and ensure equity among sectors and groups in One Health platforms at all levels; 5) support the review, update and implementation of relevant national plans, policies, legislation and programmes to integrate all dimensions of One Health, including those on biodiversity, the environment and climate change; 6) promote the national-level recognition of the human right to a clean, healthy and sustainable environment (as unanimously approved by the UN Human Rights Council in October 2021); 7) develop and ensure the inclusion of training for in-service medical, public health and veterinary professionals on the importance of and interlinkages between biodiversity conservation, links between health and the environment, how environmental destruction contributes to disease emergence, and the importance of integrating the environment sector in One Health collaborations; 8) facilitate the implementation of joint processes and workplans for One Health work; 9) conduct anthropological and participatory research to identify key risky behaviors, acceptance, and feasibility of risk mitigation measures, and appropriate alternatives, including gender-based approaches and Indigenous Peoples' knowledge; and 10) enhance private sector and NGO engagement in sustainable natural resource management, restoration activities and best practices, including climate-smart and environmentally sound healthcare (Table 6). All the average experts' agreement scores are in the top quartile (between 75 and 100% range) (Table 1, Table 2, Table 3, Table 4, Table 5, Table 6). The extended list of research priorities is available in the supplementary Tables 3–8.

**Table 6:**
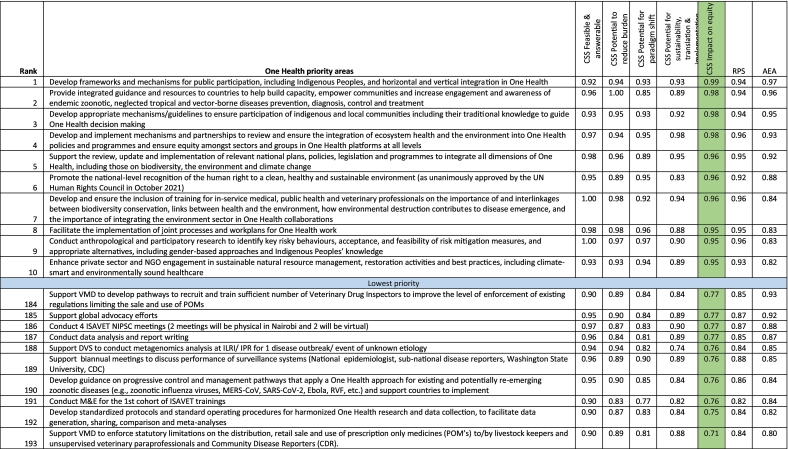
Ranking of One Health research priorities based on impact on equity Scores (partial view – top 10 and bottom 10).

Note that the complete table is available as Supplementary Table 8. CSS = Criterion-specific score; RPS = Research Priority Score; UN = United Nations; POMs = Prescription Only Medicine; NGO = Non-governmental organization; ISAVET = In-service applied Veterinary Epidemiology Training; NIPSC = National Implementation and Program Steering Committee; CDC = Centers for Disease Control and Prevention; MERS CoV = Middle East Respiratory Syndrome coronavirus; SARS CoV = Severe Acute Respiratory Syndrome coronavirus; RVF = Rift Valley fever; VMD = Veterinary Medicine Directorate; M&E = Monitoring and evaluation; DVS = Directorate of Veterinary Services; ILRI = International Livestock Research Institute; IPR = Institute of Primate Research. Average experts' agreement scores (AEA) for the top ten agenda ranged between 0.82 and 0.97, and for the bottom ten, the values ranged between 0.80 and 0.93. The table focus is on the green column.

Using this novel approach for evaluation, a number of issues and activities came to the fore in this in-exhaustive list (not in order of priority) (see supplementary Table 1 for the full list):

1. Support quarterly assessments of One Health programs implementation to document successes and identify challenges requiring enhanced training, stakeholder engagement, financing, or regulatory changes;
2. Conduct field simulation exercises at subnational level for priority zoonotic and neglected diseases affecting poor and marginalised communities;
3. Develop database of trained professionals and paraprofessionals during the duration of the previous project (the Global Health Security Agenda – Zoonotic Disease and Animal Health in Africa), their career placement and advancement to date to evaluate their ongoing contributions to the recommendations made in the previous assessments and evaluations aimed at boosting country's capacity;
4. Consider the inclusion of non-state actors like private medical and veterinary practitioners in specialised training like the in-service applied veterinary epidemiology training (ISAVET) and field epidemiology and laboratory training program (FELTP) and consider scaling up the training by linking with other partners;
5. Enhance knowledge management and information sharing through all forms of relevant communication engagements including peer and non-peer reviewed articles and targeted meetings and ‘*barazas*’ – (informal village level dissemination fora), farmers' day, stakeholders' briefings, etc., to intimate different stakeholders on progresses and successes;
6. Convert support for targeted emergency response and outbreak management as learning opportunities for engagement of trainees and future workforce through their integration into different activities;
7. Support continuous co-identification, co-planning, co-financing and co-implementation of One Health field experience learning with all other relevant One Health partners and stakeholders.

## 6 Discussion

We have utilised the CHNRI tool to generate a consultative agreement on applied and implementation research useful for and aimed at country-level development of activities, particularly in the fields of One Health. This process and outcomes are replicable and become immediately available for Kenya and other countries to prioritise activities and research questions in One Health, public health, global health, neglected zoonoses and marginalised infections in countries and regions. We find this iterative tool informative due to its simplicity, robustness, flexibility and ability to seamlessly harness consensus using a whole-of-society approach across disciplines and sectors [8,9]. Its application in generating consensus among One Health stakeholders is valid since it prevents the bias and domination of an opinion, a discipline or a field, over others. It only allows for independent yet flexible scoring by all One Health stakeholders, and the outputs are available to be combined to achieve an agreement over some composite proposals. This process further engenders intricate full or partial knowledge of all fields, and what they will be contributing, because every professional is exposed to and scores all other disciplines using an unbiased empirical process. The predefined ordinal scale allows for measurable agreement scores, it can immediately point out the level of success achieved to date in the inclusion of fields and disciplines in One Health, and areas where stakeholders need to become more open-minded. Compared to the previous priority setting approach used in Kenya to develop the past GHSA programmes, wherein key stakeholders sit in a meeting and set priorities without a formalised structure, (some of which are included in the current process (rows 124–169 in Supplementary Table 1) as ‘check questions’), and based on the comments and feedbacks from the stakeholders, and the completion rates of the tool, it becomes obvious that this co-creation process was more participatory, inclusive and speaks better to all stakeholders.

In this work, the overall RPS identified pertinent issues directly linked to mechanisms of implementations, One Health governance and legal framework, inclusiveness, coordination, anthropological and participatory research, and development and incorporation of relevant core One Health modules into higher-level training at the national level. It also recommended the development of research agenda and advocacy for matching funding for sustainability. Many of these issues have been identified in previous reports [[13], [14], [15], [16]].

Prior to this assessment, many organisations conducting activities utilizing One Health approach have excluded the private sector and sometimes the communities (particularly the indigenous and migrant populations) in their implementation strategy, but stereotypically, consider the government, implementing partners, formalised organisations, and academia [[17], [18], [19], [20]]. The outcome of this exercise clearly demonstrates the need to utilise the whole-of-society approach, leaving no-one-behind, in the implementation of One Health implementation research. The current process transitioned the respondents beyond sector-specific programs and professional silos and allowed to harness skills in co-identification and co-creating of relevant issues for co-implementation using the One Health approach in Kenya. One of the immediate outcomes was the trust in the outcome [[13], [14], [15], [16],^19^,^20^]. For instance, the private sector, the counties, the environmental sector and a few others, who originally felt left out in the fringe of implementation, now felt included as indicated in the relevant inclusion comments originating from sectors previously excluded inadvertently in One Health approach in Kenya (see below).

[*I have already forwarded the document to the Executive Committee of the private sector veterinarians. With the experience and knowledge that I have got, together with the shared report, am looking forward to meeting with the executive team, so as to internalize this GHS One Health prioritization forum (process) and come up with more inputs and areas of engagements beyond what I already forwarded in the report. On behalf of the private sector, we felt privileged to be part of the team that participated in contributing to the above activity. We look forward to a continuous partnership with your organization so as to bring in the input of self-employed veterinarians who are always on the first line of Animal Health Services Provision for the farmers all over the country*]. – Extracted from an email from the Vice Chair of the Private Sector Veterinarians.

[*We appreciate the all-inclusive approach that brought together all relevant stakeholders in One Health, towards prioritization of GHSA activities. This is commendable! From the preliminary results, it truly reflects the aspirations of the stakeholders. This approach will go a long way in helping the Counties (subnational government) to align their priorities and justify (based on facts) the inclusion of complimentary One Health activities in the county integrated development plan (CIDP)*]. – Extracted from an email from a county Epidemiologist.

The private sector has resources and expertise that are key in strengthening of global health security, their exclusion (including the environmental sector) remains a major impediment in the successful programmatic implementation using a One Health approach [15,21,22]. Similarly, many implementing partners developed their lists of One Health activities, and possibly shared them later with other partners but not necessary for co-creation and joint implementation. This process has identified joint processes in work planning and implementation of activities as very relevant for a successful One Health engagement in Kenya. This assessment provides a template for sustainability of programmes that utilise One Health approach and summons the whole of society approach, inclusive of the funders and private sector engagement (PSE) and beneficiary communities (Table 1).

Identifying and ranking research priorities for One Health programming is critical in resource limited settings where the government heavily relies on other partners' funding. There are also numerous competing interests in the devolved health and agriculture sectors in Kenya hence the need for justifiable and cost-effective investment decision(s) using empirical criteria to prioritise activities and ultimately gain buy-in from multiple stakeholders. Furthermore, the application of One Health within countries often comes with unintended consequences including but not limited to duplication of implementation of activities by partners across the countries, particularly where coordination is lacking, and monitoring and evaluation of One Health approach is not regularly implemented. The consultative nature of this process, provides opportunities for collaboration and shared knowledge among implementing partners for co-identification, co-creation, co-financing and cost-effective joint implementation of relevant One Health projects. Sometimes, implementing organisations, government entities, decision-makers and policy implementers also need to identify their strengths, weaknesses, opportunities and threats based on the rich information they receive from the beneficiary communities and other stakeholders [23]. Funding agencies have identified the basis and benefits of such co-creation, which allows end-users to define a problem collaboratively, collectively identify and design new or refine existing solutions, build consensus around action, and refine plans to move forward with programs and projects [24,25]. Issues that will support sustainability and translational research have been identified in this work, and future implementers and policy makers should consider benchmarking them in developing future research agenda in low- and middle-income countries.

## 7 Limitations

Although the questionnaire was comprehensive and represented a matrix of ideas from several consultative empirical engagements, assessors often complained about the length of the spreadsheet. Specifically, each assessor needed to score 965 times after a deep reflection on each question and the matching criterion. Assessors spent on average, a minimum of 150 min (2 h, 30 min) and up to 330 min (5 h, 30 min) in doing thorough scoring. This length allows room to sometimes become flippant and not deeply reflective as boredom and fatigue set in. To reduce the likelihood of this effect, which may introduce some degree of error and bias into the study, we partitioned the 193 questions into 9 sections, encouraged participants (assessors) to undertake the responsibility in piecemeal and take breaks in-between sections of the assessment. Whereas some assessors suggested that the tool may be divided into specific thematic areas to allow for focused evaluation, or to send the questionnaires in bits covering each section, these opinions were deemed detrimental to the spirit of One Health consensus building process and may retrogress to the ‘*silo mentality*’ during project implementation. The investment of time and efforts have been identified as a challenge in the co-creation process in health-related research by other researchers [26]. For instance, if the questions are sent in sections, after filling one or two rounds, the assessors may be unwilling to continue the process.

Furthermore, the individual ranking of ‘1.0’, ‘0.5’, ‘0.0’ and blank appears subjective using the standardized tool. It particularly posed a degree of difficulty especially for researchers and implementers from different backgrounds and perspectives, who are were unfamiliar with the use of several key concepts and terminologies in One Health. However, during the consultative workshop, there was constant engagement and facilitated guidance by the facilitators and these engagements during the stakeholders' consultation improved the stakeholders' context and scoring, the off-site respondents' ability to contextualise and respond concisely to the questions may have been limited.

Finally, it was difficult to cluster the professionals per specific discipline because a number have cross disciplines, fields and sectors, e.g. a veterinarian with MSc in virology and PhD in epidemiology, a medical doctor who ended up in policy and an environmentalist who is now a One Health champion. Some of the experts who attended the stakeholders' consultation meeting had earlier filled the tool sent through the email and were not under obligation to fill it again.

## 8 Implications for policy, practice, and research

This study had systematically co-created and generated a list of prioritised One Health areas in Kenya for potential research and implementation that should enhance the prevention, detection and response to emerging public health threats in line with the revised JEE v3.0 [2] and GHSA action packages of AMR, Zoonoses, Real-time surveillance, Biosafety and Biosecurity, workforce development, emergency operation centres and laboratory diagnostics. Its outcome should inform strategic implementation, prudent economic investment, acceptability from the whole of society, and outlines shared responsibilities by implementers, funders, government, and other stakeholders to address aspirations in the different instruments including but not limited to JEE v3.0, PVS, SDGs. While the outcome of research priority scores (RPS) can be used for focused implementation, the flexibility of utilizing the criterion specific scores (CSS) for priority setting should also appeal to policy makers and implementers. Researchers in the fields utilizing One Health approach also have immediate opportunity to pick areas of research that are impact-driven for the society. Furthermore, this work should assist in boosting coordinated national surveillance and risk management capacity, grounded in appropriate regulatory frameworks, and encourage notification to and alignment with regional and global frameworks and existing priority disease programmes.

Finally, this study bolsters the multidisciplinary synergy using a multicriteria One Health decision making processes to identify areas of multisectoral convergence in global health security that will guide priority areas of One Health investment, cost effective and sustainable implementation between the private sector, partners and government of Kenya.

## Author contributions

FOF conceptualized the study, curated the questions to develop the customized CHNRI tool and conducted the data analysis. MN and RWS reviewed the tool, assisted in stakeholders' identification, collected the data and assisted in data analysis and presentation. All other authors contributed scores, participated in validation and provided significant intellectual inputs in review and editing.

## Ethical clearance and informed consent

No ethical issue arose in the process of implementing this research. However, all participants consented individually, were nominated by their offices, or sought clearance and permission from their offices to participate in the study. Each participant willingly contributed to filling the tool and availed themselves for follow up discussions. This work did not involve any invasive or privacy intrusion methods on the participants.

## Declaration of Competing Interest

The authors declare no conflicts of interest.

Fasina FO for all authors.

## Data Availability

Data will be made available on request.

## Appendix A Supplementary data

Supplementary material 1 Image 1

Supplementary material 2 Image 2

Supplementary material 3 Image 3

Supplementary material 4 Image 4

Supplementary material 5 Image 5

Supplementary material 6 Image 6

Supplementary material 7 Image 7

Supplementary material 8 Image 8
