# Supplementary material for: Co-creation and priority setting for applied and implementation research in One Health: Improving capacities in public and animal health systems in Kenya
Source: One Health. 2022 Nov 17;15:100460. doi: 10.1016/j.onehlt.2022.100460 (PMC9754982; doi:10.1016/j.onehlt.2022.100460)
Supplement: Supplementary file 2 — Supplementary material 2 [file mmc2.docx]

**Supplementary Table 2: Library of Terms**

**Co-creation and priority setting for applied and implementation research in One Health: improving country’s capacities in public and animal health systems in Kenya**

|  |  |
| --- | --- |
| Term or acronym | **Full name or explanation** |
| AEA | Average Experts’ Agreement |
| AFROHUN | Africa One Health University Network |
| AMR | Antimicrobial resistance |
| ASALs | Arid and semi-arid lands |
| CAVS | College of Agriculture and Veterinary Sciences |
| CBOs | Community-based organizations |
| CDC | Centers for Disease Control and Prevention |
| CDRs | Community Disease Reporters |
| CDVSs | County Directorate of Veterinary Services |
| CHNRI | Child Health and Nutrition Research Initiative |
| CSS | Criterion-specific score |
| DVS | Directorate of Veterinary Services |
| ECTAD | Emergency Center for Transboundary Animal Diseases, |
| FAO | Food and Agriculture Organization of the United Nations |
| GHSA | Global Health Security agenda |
| GHSP | Global Health Security programs |
| FBOs | Faith-based organizations |
| RPS | Research Priority Scores |
| H-NAPs | Health National Adaptation Plans |
| IHR (2005) | International Health Regulations (2005) |
| ILRI | International Livestock Research Institute |
| INGOs | International non-governmental organizations |
| IPC | Infection prevention and control |
| IPR | Institute of Primate Research |
| ISAVET | In-service applied Veterinary Epidemiology Training |
| JEE | Joint External Evaluation |
| MCM OT | One Health multisectoral One Health coordination mechanisms operation tool |
| M&E | Monitoring and evaluation |
| MEA | Multilateral environmental agreements |
| MERS CoV | Middle East Respiratory Syndrome coronavirus |
| MoALF&C | Ministry of Agriculture, Livestock, Fisheries and Co-operatives |
| MoH | Ministry of Health |
| NAPHS | National Action Planning for Health Security |
| NDC | Nationally Determined Contributions |
| NGOs | Non-governmental organizations |
| NIPSC | National Implementation and Program Steering Committee |
| NTDs | Neglected tropical diseases |
| OHHLEP | One Health High-Level Expert Panel |
| OH JPA | One Health Joint Plan of Action |
| PVS | Performance of Veterinary Services |
| POMs | Prescription only medicines |
| PPP | Public Private Partnership |
| PZDs | Priority zoonotic diseases |
| RVF | Rift Valley fever |
| RVILs | Regional Veterinary Investigation Laboratories |
| SARS CoV | Severe Acute Respiratory Syndrome coronavirus |
| SDGs | Sustainable Development Goals |
| UNON | United Nations Office in Nairobi |
| USAID | United States Agency for International Development |
| VBDs | Vector borne diseases |
| VMD | Veterinary Medicine Directorate |
| VSVPA | Veterinary Surgeons and Veterinary Paraprofessional Act |
| WHE | World Health Emergencies |
| WHO | World Health Organization |
| WOAH | World Organization for Animal Health |
| ZDU | Zoonotic Disease Unit |
