# Supplementary material for: Co-creation and priority setting for applied and implementation research in One Health: Improving capacities in public and animal health systems in Kenya
Source: One Health. 2022 Nov 17;15:100460. doi: 10.1016/j.onehlt.2022.100460 (PMC9754982; doi:10.1016/j.onehlt.2022.100460)
Supplement: Supplementary file 5 — Supplementary material 5 [file mmc5.docx]

***Supplementary Table 5. One Health Research Ranking Based on Potential to reduce burden Scores***

|  | Areas of One Health research | CSS Feasible & answerable | CSS Potential to reduce burden | CSS Potential for paradigm shift | CSS Potential for sustainability, translation & implementation | CSS Impact on equity | RPS |
| --- | --- | --- | --- | --- | --- | --- | --- |
| 1 | Build collaborative predictive epidemic intelligence systems (at national, regional, and global levels) to identify high-risk interfaces and hot spots for spillover, incorporating relevant environment and climate data and data on establishment of reservoirs and vector species in new geographic areas | 0.98 | 1.00 | 0.98 | 0.92 | 0.89 | 0.95 |
| 2 | Identify actionable solutions/innovative procedures to address the limitation to adopt recomended IPC, including proper vaccination, biosecurity, and food handling practices for identified diseases | 0.98 | 1.00 | 0.92 | 0.91 | 0.87 | 0.94 |
| 3 | Provide integrated guidance and resources to countries to help build capacity, empower communities and increase engagement and awareness of endemic zoonotic, neglected tropical and vector-borne diseases prevention, diagnosis, control and treatment | 0.96 | 1.00 | 0.85 | 0.89 | 0.98 | 0.94 |
| 4 | Provide technical support and develop training programmes to ensure all countries can conduct food safety risk analysis under a One Health approach and a food systems lens | 0.96 | 1.00 | 0.94 | 0.96 | 0.88 | 0.95 |
| 5 | Support countries to Include or strengthen the One Health approach in the food safety incident and emergency response plans | 0.95 | 1.00 | 0.93 | 0.95 | 0.89 | 0.95 |
| 6 | Leverage innovations and new technologies in disease surveillance, rapid response, and control | 0.93 | 1.00 | 0.94 | 0.88 | 0.92 | 0.93 |
| 7 | Engage with local communities including Indigenous Peoples, to identify sustainable solutions, nature-based where applicable, for the prevention and control of emerging and re-emerging zoonotic diseases | 0.93 | 1.00 | 0.96 | 0.92 | 0.92 | 0.95 |
| 8 | Conduct hotspot mapping of PZDs (e.g. Rabies, RVF, Anthrax, Brucellosis), and other Economically Important Livestock diseases to develop animal health diseases risk map/atlas | 0.96 | 0.99 | 0.94 | 0.92 | 0.88 | 0.94 |
| 9 | Renovation and equipping of border inspection posts | 0.96 | 0.99 | 0.80 | 0.85 | 0.88 | 0.90 |
| 10 | Raise awareness with key stakeholders about the benefits of healthy ecosystems, identified risk factors and drivers, as well as solutions for risk mitigation and spillover prevention that are nature-based where applicable, acceptable, and sustainable | 0.99 | 0.99 | 0.93 | 0.90 | 0.94 | 0.95 |
| 11 | Support countries to strengthen legislation and programmes for monitoring and surveillance for foodborne diseases, including surveillance for antimicrobial resistant foodborne pathogens. | 0.95 | 0.99 | 0.90 | 0.94 | 0.89 | 0.93 |
| 12 | Support balanced, functional, well-represented national inter-agency coordination mechanisms, and One Health approaches to AMR National Action Plan (NAP) implementation | 1.00 | 0.98 | 0.96 | 0.96 | 0.84 | 0.95 |
| 13 | Promote country ownership and galvanize international collaboration to support One Health policies and legislation for endemic zoonotic, neglected tropical and vector-borne diseases | 1.00 | 0.98 | 0.92 | 0.85 | 0.92 | 0.93 |
| 14 | Provide countries with operational tools and resources for integrated multisectoral surveillance and mapping of risk areas for endemic zoonotic, neglected tropical and vector-borne diseases from national to local levels | 0.96 | 0.98 | 0.90 | 0.91 | 0.88 | 0.93 |
| 15 | Facilitate the implementation of joint processes and workplans for One Health work | 0.98 | 0.98 | 0.96 | 0.88 | 0.95 | 0.95 |
| 16 | Provide resources and support to countries to implement proven disease control strategies, as for example proposed by the Zero by Thirty: the Global Strategic Plan to Eliminate Human Deaths from Dog Mediated Rabies by 2030, as a way of operationalizing a One Health approach | 0.94 | 0.98 | 0.87 | 0.89 | 0.89 | 0.92 |
| 17 | Promote, inform and support the sound management of chemicals and waste, including wastewater, and the prevention, reduction and control of pollution into air, water and soil, in order to minimize threats to the health of ecosystems, animals, plants and people | 0.93 | 0.98 | 0.94 | 0.89 | 0.92 | 0.93 |
| 18 | Support (technical and logistics) 2 outbreak After Action Reviews and use the information to improve future response to outbreaks/ prevention of similar outbreaks | 0.92 | 0.98 | 0.94 | 0.94 | 0.89 | 0.93 |
| 19 | Establish effective risk analysis and risk management protocol at DVS and train CDVS for implementation of identified mitigation measures | 0.96 | 0.98 | 0.93 | 0.96 | 0.88 | 0.94 |
| 20 | Develop and ensure the inclusion of training for in-service medical, public health and veterinary professionals on the importance of and interlinkages between biodiversity conservation, links between health and the environment, how environmental destruction contributes to disease emergence, and the importance of integrating the environment sector in One Health collaborations | 1.00 | 0.98 | 0.92 | 0.94 | 0.96 | 0.96 |
| 21 | Support countries to provide access to quality vaccines, medicines and basic water, sanitation, and hygiene services (WASH) services, including agricultural water use waste management including animal waste and carcass disposal and training of communities across sectors to address endemic zoonotic, neglected tropical and vector-borne diseases | 0.95 | 0.97 | 0.85 | 0.81 | 0.91 | 0.90 |
| 22 | Identify One Health research gaps and priorities, develop a research agenda and advocate for funding to find sustainable solutions to reduce the risk of disease emergence | 1.00 | 0.97 | 0.98 | 0.96 | 0.86 | 0.96 |
| 23 | Identify drivers and indicators to monitor their impacts on zoonotic disease emergence, re-emergence, and spread, including those that can lead to increased interfaces or disruptions of natural host-pathogen dynamics | 0.96 | 0.97 | 0.93 | 0.93 | 0.84 | 0.93 |
| 24 | Promote effective communication structures and information and data sharing systems across organisations, sectors, and society | 0.93 | 0.97 | 0.94 | 0.90 | 0.91 | 0.93 |
| 25 | Conduct anthropological and participatory research to identify key risky behaviours, acceptance, and feasibility of risk mitigation measures, and appropriate alternatives, including gender-based approaches and Indigenous Peoples’ knowledge | 1.00 | 0.97 | 0.97 | 0.90 | 0.95 | 0.96 |
| 26 | Development of disease control strategies for more PZDs not yet covered | 0.98 | 0.97 | 0.86 | 0.89 | 0.87 | 0.91 |
| 27 | Develop/update guidelines and innovative approaches for best practice for harmonizing One Health concepts into harmonized joint food safety risk analyses in the context of the existing food system | 0.97 | 0.97 | 0.93 | 0.96 | 0.87 | 0.94 |
| 28 | Provide technical supporting tools (checklists, legal frameworks evaluations, etc.) and strengthen capacity building for countries in the development of food control systems and regulatory frameworks that incorporate more systematically the regulatory frameworks under a One Health approach | 0.98 | 0.97 | 0.89 | 0.93 | 0.89 | 0.93 |
| 29 | Support national level and subnational surveillance officers to increase coordination of surveillance activities across the country through provision of weekly feedback reports to counties and bi-annual meetings | 0.96 | 0.97 | 0.89 | 0.91 | 0.90 | 0.93 |
| 30 | Develop operational tools and resources to conduct targeted One Health surveillance at human-animal-ecosystem interfaces and mechanism for multisectoral data sharing as per Tripartite/UNEP/OHHLEP guidance, and supported by robust regulatory frameworks | 0.98 | 0.97 | 0.93 | 0.89 | 0.87 | 0.93 |
| 31 | Facilitate inclusion and training of private veterinary practitioners under CDVS supervision for implementation of standardised disease control and eradication strategies | 0.98 | 0.97 | 0.90 | 0.92 | 0.88 | 0.93 |
| 32 | Develop and promote the implementation of joint guidelines for the environmentally sound management of public health, medical and veterinary operations and their waste | 0.95 | 0.97 | 0.91 | 0.88 | 0.89 | 0.92 |
| 33 | Targeted human capacity trainings for subnational level staff in the application of strategies/control documents | 0.99 | 0.97 | 0.87 | 0.93 | 0.91 | 0.93 |
| 34 | Conduct/support laboratory testing of collected samples at CVL/KEMRI (collect samples and take them to CVL/KEMRI for testing) | 0.94 | 0.97 | 0.85 | 0.91 | 0.84 | 0.90 |
| 35 | Provide and preposition outbreak investigation kits in selected RVILs (Garissa, Mariakani, Kericho, Eldoret, Nakuru, Karatina) | 1.00 | 0.97 | 0.83 | 0.89 | 0.82 | 0.90 |
| 36 | Develop mechanisms to support an overarching One Health governance and legal framework | 1.00 | 0.96 | 0.96 | 0.96 | 0.93 | 0.96 |
| 37 | Develop guidance to conduct coordinated and systematic data collection, operational and behavioural research, and risk assessment on the drivers, processes, and pathways for zoonotic disease emergence, spread and persistence as well as to characterize intact resilient healthy ecosystems and their effect on disease prevention and support countries in the implementation | 0.98 | 0.96 | 0.87 | 0.88 | 0.80 | 0.90 |
| 38 | Support countries to operationalize existing global strategies on zoonotic diseases and ensure synergy and cohesiveness at national level | 1.00 | 0.96 | 0.87 | 0.92 | 0.86 | 0.92 |
| 39 | Support countries to build the investment case and develop sustainable financing and governance mechanisms for cost-effective endemic zoonotic, neglected tropical and vector-borne diseases control through implementation of One Health principles | 0.99 | 0.96 | 0.94 | 0.87 | 0.91 | 0.94 |
| 40 | Develop a One Health indicator framework to monitor human, wildlife, domestic animal, vector, and environmental health, including in intact resilient healthy ecosystems to establish baselines, and support countries to monitor changes over time/along development gradients | 0.96 | 0.96 | 0.94 | 0.89 | 0.86 | 0.92 |
| 41 | Support countries to strengthen disease reporting and integrated data collection, information sharing and outbreak response to build multisectoral, One Health coordinated national surveillance and risk management capacity, grounded in appropriate regulatory frameworks, and encourage notification to and alignment with regional and global frameworks and existing priority disease programmes | 1.00 | 0.96 | 0.93 | 1.00 | 0.92 | 0.96 |
| 42 | Provide technical support and capacity development activities for countries in targeted areas | 0.96 | 0.96 | 0.96 | 0.93 | 0.86 | 0.94 |
| 43 | Support ( technical and logistics) World Rabies Day (WRD) events including providing logistics for awareness creation like radio hot posts, printing of awareness materials, logistics for vaccinations and neautering, and participation in WRD conference | 0.96 | 0.96 | 0.84 | 0.91 | 0.88 | 0.91 |
| 44 | One Health framework for food safety, capturing the pathways and connections in food safety activities that lead to positive and negative health outcomes in humans, animals and environment -throughout the food supply chain | 0.98 | 0.96 | 0.92 | 0.88 | 0.87 | 0.92 |
| 45 | Identify and quantify the main anthropogenic factors leading to environmental degradation that negatively impact the health of ecosystems, animals, plants and people | 0.96 | 0.96 | 0.92 | 0.92 | 0.93 | 0.94 |
| 46 | Rehabilitate the 6 regional laboratories | 0.95 | 0.96 | 0.85 | 0.87 | 0.87 | 0.90 |
| 47 | Strengthen information, awareness and control of vector- and rodent-borne diseases and their specific threat to urban centres | 0.95 | 0.96 | 0.84 | 0.82 | 0.84 | 0.88 |
| 48 | Ensure effective management of the AMR Multi-Partner Trust Fund (MPTF) | 0.92 | 0.96 | 0.86 | 0.88 | 0.88 | 0.90 |
| 49 | Support countries to strengthen surveillance systems for estimating and sharing data on the burden of foodborne illness and attributing illnesses to specific food sources, to better target prevention and control measures under a One Health approach | 1.00 | 0.96 | 0.89 | 0.96 | 0.89 | 0.94 |
| 50 | Support the review, update and implementation of relevant national plans, policies, legislation and programmes to integrate all dimensions of One Health, including those on biodiversity, the environment and climate change | 0.98 | 0.96 | 0.89 | 0.95 | 0.96 | 0.95 |
| 51 | Support development of a structured risk analysis process to facilitate decision-making process to prevent the introduction and spread of diseases | 0.94 | 0.96 | 0.85 | 0.92 | 0.86 | 0.91 |
| 52 | Promote the transition towards sustainable, climate-smart, agroecological approaches to agriculture, aquaculture livestock production and non-timber forest products, including through regulations, to reduce risks to the health of the environment, animals, plants and people | 0.96 | 0.96 | 0.96 | 0.82 | 0.87 | 0.91 |
| 53 | Support training & collaboration with law enforcement and border control authorities to tighten up on illegal internal and cross-border movement of live animals and animal products | 0.94 | 0.96 | 0.88 | 0.92 | 0.81 | 0.90 |
| 54 | Support continuous training and retraining of county animal health and wildlife/tourism staff on KABS for improved animal disease reporting | 0.97 | 0.96 | 0.92 | 0.94 | 0.88 | 0.93 |
| 55 | Support implementation of LITS to assist in the management of the risk posed by internal animal movements | 0.92 | 0.96 | 0.91 | 0.90 | 0.81 | 0.90 |
| 56 | Develop a road map for implementation of actvities to address the major gaps identified in the previous implementation to date | 0.98 | 0.96 | 0.86 | 0.96 | 0.86 | 0.92 |
| 57 | Facilitate One Health capacity building, including workforce development in all relevant sectors | 0.98 | 0.96 | 0.91 | 0.91 | 0.94 | 0.94 |
| 58 | Design a monitoring and evaluation framework for continuous improvement of the organisations’ and countries’ One Health actions, performances, and capacities | 0.98 | 0.96 | 0.87 | 0.96 | 0.92 | 0.94 |
| 59 | Establish financial needs to build One Health capacity at national and subnational level | 0.91 | 0.96 | 0.89 | 0.82 | 0.89 | 0.89 |
| 60 | Support the implementation of the Convention on Biological Diversity Global Action Plan on Biodiversity and Health, and related action plans and operational frameworks | 0.93 | 0.95 | 0.93 | 0.83 | 0.86 | 0.90 |
| 61 | Develop and update standards and technical advice on global best practice | 1.00 | 0.95 | 0.87 | 0.91 | 0.88 | 0.92 |
| 62 | Support countries to strengthen the capacity to identify and evaluate new and emerging food safety issues, including new and emerging risk arising at human-animal-plant-environment interface | 0.98 | 0.95 | 0.89 | 0.94 | 0.85 | 0.92 |
| 63 | Support the inter-related structures to strengthen the accountability and global governance of AMR | 0.95 | 0.95 | 0.91 | 0.88 | 0.83 | 0.91 |
| 64 | Train health officers at national and sub-national levels from human, animal and environmental health sectors on Joint Risk Assessment (JRA). Target counties include (West Pokot, Kisumu, Kakamega, Kajiado, Migori,Bomet, Garissa, Mandera, Moyale, Wajir, Nyandarua, Muran'ga, Kiambu, Nairobi, Baringo, Turkana, Siaya, Narok, Isiolo, Nyeri, Nakuru, Busia,Kitui,Lamu,Samburu,Marsabit) | 0.94 | 0.95 | 0.96 | 0.93 | 0.89 | 0.94 |
| 65 | Support VMD to convene workshops for stakeholder engagement towards review of the legal framework on matters regading regulation of veterinary medicines | 0.97 | 0.95 | 0.86 | 0.90 | 0.80 | 0.90 |
| 66 | Develop appropriate mechanisms/guidelines to ensure participation of indigenous and local communities including their traditional knowledge to guide One Health decision making | 0.93 | 0.95 | 0.93 | 0.92 | 0.98 | 0.94 |
| 67 | Establish linkages among disease databases and environment databases to support risk modelling, shared information and informed/science-based decision and policy making | 0.98 | 0.95 | 0.92 | 0.88 | 0.87 | 0.92 |
| 68 | Support harmonization of veterinary policies & legislation with other existing laws and regulations | 1.00 | 0.95 | 0.90 | 0.98 | 0.84 | 0.93 |
| 69 | Support improvement of real-time 4-way linkage and information management in veterinary and public health laboratories and epidemiology units to enhance early detection, identification, response and information flow within the system. | 1.00 | 0.95 | 0.96 | 0.96 | 0.90 | 0.95 |
| 70 | Support a 5-year cycle meeting of intensive planning exercise to align operational needs with national policies for disease control and other national policies to create enabling environment for resource mobilization | 1.00 | 0.95 | 0.89 | 0.93 | 0.89 | 0.93 |
| 71 | Conduct Biorisk Assessment and LMT safety assessment at national referral (CVL, FMD)) and subnational laboratories (RVILs- Eldoret, Garissa, Kericho, Karatina, Mariakani, Nakuru) | 1.00 | 0.95 | 0.87 | 0.89 | 0.87 | 0.91 |
| 72 | Support and promote the next generation of One Health practitioners, researchers and technical officers | 0.98 | 0.95 | 0.96 | 0.88 | 0.85 | 0.92 |
| 73 | Strengthen regional collaboration on AMR | 0.97 | 0.95 | 0.90 | 0.94 | 0.88 | 0.93 |
| 74 | Support countries to include endemic zoonotic, neglected tropical and vector-borne diseases when establishing national One Health mechanisms and One Health disease control strategic plans | 1.00 | 0.95 | 0.93 | 0.95 | 0.90 | 0.94 |
| 75 | Define planning mechanisms for One Health coordination, including collaborative governance mechanisms, policies and legal frameworks, and capacity building strategies applicable at national and subnational level | 0.96 | 0.95 | 0.93 | 0.90 | 0.86 | 0.92 |
| 76 | Build evidence base for One Health approach in reducing disease burden and socioeconomic impact of endemic zoonotic, neglected tropical and vector-borne diseases across relevant sectors, from global to national levels | 0.95 | 0.94 | 0.94 | 0.91 | 0.91 | 0.93 |
| 77 | Support countries to conduct pathogen surveillance at the human-animal-environment interface through technical network support predictive epidemic intelligence, including the monitoring of trends in pathogen mutation and evolution and microbial diversity in wildlife as well as prediction of zoonotic or spillover potential of novel pathogens | 0.98 | 0.94 | 0.94 | 0.90 | 0.80 | 0.91 |
| 78 | Provide guidance and tools for One Health transparent and trusted collaborative governance, mechanisms, policies, and regulatory frameworks | 0.93 | 0.94 | 0.89 | 0.88 | 0.87 | 0.90 |
| 79 | Use pandemic risk assessment approaches (e.g., WHO's Tool for Influenza Pandemic Risk Assessment - TIPRA, molecular risk assessment, FAO's EMPRES-i Genetic Module) to proactively identify pre-pandemic vaccine candidates for existing zoonotic pathogens to inform vaccine production | 0.97 | 0.94 | 0.94 | 0.87 | 0.84 | 0.91 |
| 80 | Support faculty training and the development of core modules on public health in the environment academic curricula. | 1.00 | 0.94 | 0.96 | 0.99 | 0.88 | 0.95 |
| 81 | Procure maintenance and calibration services for selected biosafety equipment at CVL and selected sub-national veterinary laboratories (Kericho) | 1.00 | 0.94 | 0.83 | 0.85 | 0.82 | 0.89 |
| 82 | Support linkage between VMD and the CDVSs to provide oversight on agrovets | 0.98 | 0.94 | 0.84 | 0.84 | 0.82 | 0.88 |
| 83 | Provide guidance for the management of food safety risks under the One Health approach | 0.96 | 0.94 | 0.93 | 0.93 | 0.93 | 0.94 |
| 84 | Design pilot interventions that include innovative procedures to increase the adoption of key good practices at selected dairy and beef value chain nodes | 1.00 | 0.94 | 0.86 | 0.91 | 0.85 | 0.91 |
| 85 | Manage databases and tools to collect, improve access to and interpret relevant food safety data and other information, including water, food, and wildlife meat | 0.98 | 0.94 | 0.88 | 0.83 | 0.87 | 0.90 |
| 86 | Develop and implement mechanisms and partnerships to review and ensure the integration of ecosystem health and the environment into One Health policies and programmes and ensure equity amongst sectors and groups in One Health platforms at all levels | 0.97 | 0.94 | 0.95 | 0.98 | 0.98 | 0.96 |
| 87 | Translate environmental knowledge and data to improve policies and legislation and propose practical solutions to prevent and mitigate health threats at the interfaces | 0.97 | 0.94 | 0.92 | 0.92 | 0.94 | 0.94 |
| 88 | Develop and roll out a national Environment Sector Needs Assessment Tool to benchmark institutional and individual capacity to participate interoperably in all aspects of One Health in support of FTP-WEBE (Field Training Programme for Wildlife, Environment, Biodiversity and Ecosystems Professionals) | 0.97 | 0.94 | 0.93 | 0.88 | 0.94 | 0.93 |
| 89 | Support human resource development through evaluation of training quality management systems (TQMs) and piloting of TQMS on the Virtual Learning Centre (VLC) as informed by regional QA programme | 0.94 | 0.94 | 0.92 | 0.96 | 0.85 | 0.92 |
| 90 | Develop a prioritized research agenda to provide direction for investment | 1.00 | 0.94 | 0.92 | 0.92 | 0.87 | 0.93 |
| 91 | Promote One Health cross-sectoral collaboration and partnerships, including Public Private Partnership (PPP) | 0.96 | 0.94 | 0.96 | 0.96 | 0.85 | 0.94 |
| 92 | Develop frameworks and mechanisms for public participation, including Indigenous Peoples, and horizontal and vertical integration in One Health | 0.92 | 0.94 | 0.93 | 0.93 | 0.99 | 0.94 |
| 93 | Generate mechanisms for joint funding and resource mobilization | 0.94 | 0.94 | 0.85 | 0.91 | 0.84 | 0.90 |
| 94 | Print and distribute Kenya Biorisk management Curriculum (KBRMC) | 0.96 | 0.94 | 0.93 | 0.87 | 0.80 | 0.90 |
| 95 | Support DVS to conduct metagenomics analysis at ILRI/ IPR for 1 disease outbreak/ event of unknown etiology | 0.94 | 0.94 | 0.82 | 0.74 | 0.76 | 0.84 |
| 96 | Support the integration of health and environment considerations and including risks in impact assessments and performance standards of the International Finance Corporation (IFC) and other financial institutions | 0.96 | 0.93 | 0.91 | 0.92 | 0.89 | 0.92 |
| 97 | Conduct review of country status on implementation of the National Rabies Elimination Strategy | 1.00 | 0.93 | 0.83 | 0.92 | 0.89 | 0.92 |
| 98 | Establish standards for the management of ecosystem processes at all levels to support resilience including mainstreaming habitat degradation prevention and biodiversity protection in food systems to maximize co-benefits | 0.91 | 0.93 | 0.94 | 0.79 | 0.88 | 0.89 |
| 99 | Enhance One Health related research for the transmission of foodborne pathogens and food contaminants at the human-animal-plant-environment interface | 1.00 | 0.93 | 0.95 | 0.92 | 0.85 | 0.93 |
| 100 | Support quarterly OH update and coordination meetings between the Zoonotic Disease Unit (ZDU) and 5 County One Health Units (COHU)- ZTWG | 1.00 | 0.93 | 0.95 | 0.90 | 0.86 | 0.93 |
| 101 | OIE endorsement program on monitoring and support of Kenya rabies control program/strategy | 0.97 | 0.93 | 0.80 | 0.88 | 0.78 | 0.87 |
| 102 | Incorporate land use planning in health and biodiversity risks assessments and vice versa | 0.89 | 0.93 | 0.90 | 0.81 | 0.88 | 0.88 |
| 103 | Conduct 4 ISAVET ITWG meetings (2 meetings will be physical in Nairobi and 2 will be virtual) | 0.96 | 0.93 | 0.83 | 0.86 | 0.80 | 0.88 |
| 104 | Support joint coordination platform with National Disaster Operation Centre and Emergency Operations Centre | 0.97 | 0.93 | 0.90 | 0.93 | 0.87 | 0.92 |
| 105 | Support implementation the MALF Quality Policy of Kenya by appointing dedicated Quality Managers at all public veterinary laboratories giving priority for ISO 17205 accreditation at the CVL and all RVILs | 0.96 | 0.93 | 0.90 | 0.96 | 0.89 | 0.93 |
| 106 | Build cold chain facility and support with equipment provision to the 6 regional laboratories | 0.96 | 0.93 | 0.77 | 0.85 | 0.91 | 0.88 |
| 107 | Map out, review and revise existing tools aiming to improve multisectoral collaboration (e.g., IHR-PVS National Bridging Workshops (NBW) and the Tripartite operational tools under the Tripartite zoonotic guide) ensuring environmental considerations are well integrated in them, and develop new tools to support integration of environment aspects of One Health, as needed | 1.00 | 0.93 | 0.93 | 0.95 | 0.89 | 0.94 |
| 108 | Conduct a gap analysis based on the results of the workforce profiling report. | 0.98 | 0.93 | 0.92 | 0.90 | 0.93 | 0.93 |
| 109 | Identify incentives and co-benefits, and raise awareness of the central role of the environmental sector about the importance of their participation and role in One Health | 0.97 | 0.93 | 0.94 | 0.96 | 0.92 | 0.94 |
| 110 | Develop a One Health Introductory course that can be delivered simultaneously to in-service professionals from all One Health sectors (health, animal health, environment) and serves as a prerequisite for ISAVET, FETP, FETPV, and FTP-WEBE training | 0.96 | 0.93 | 0.95 | 0.91 | 0.89 | 0.93 |
| 111 | Develop and train on protocols and data collection tools for collection of samples and post-market surveillance data | 0.96 | 0.93 | 0.87 | 0.90 | 0.78 | 0.89 |
| 112 | Convene relevant sectors to facilitate integrated land and sea use planning that incorporates human, animal and environmental co-benefits and yields sustainable land and water management | 0.95 | 0.93 | 0.98 | 0.99 | 0.95 | 0.96 |
| 113 | Enhance private sector and NGO engagement in sustainable natural resource management, restoration activities and best practices, including climate-smart and environmentally sound healthcare | 0.93 | 0.93 | 0.94 | 0.89 | 0.95 | 0.93 |
| 114 | Conduct data analysis based on previous GHSA supported surveillance on AMR to support development of policy briefs | 1.00 | 0.93 | 0.89 | 0.90 | 0.79 | 0.90 |
| 115 | Develop joint information management systems and analytical tools integrating ecosystem, environmental, animal and human health knowledge and data | 0.99 | 0.93 | 0.99 | 0.92 | 0.89 | 0.94 |
| 116 | Define One Health institutional and workforce capacities and develop methodologies and tools to assess national One Health performances and identify needs | 0.97 | 0.93 | 0.91 | 0.90 | 0.87 | 0.92 |
| 117 | Support and link activities to Nationally Determined Contributions (NDCs), National Biodiversity Strategies and Action Plans (NBSAPs) other commitments under multilateral environmental agreements (MEAs) and Health National Adaptation Plans (H-NAPs) commitments made by national governments to address climate change and environmental degradation | 0.90 | 0.93 | 0.95 | 0.87 | 0.81 | 0.89 |
| 118 | Support countries to conduct joint One Health Risk Assessments and mapping leading to evidence-based and targeted risk management and communication | 1.00 | 0.93 | 0.96 | 0.96 | 0.89 | 0.95 |
| 119 | Define processes and develop methodologies for assessing countries’ vulnerabilities to One Health challenges, and link with appropriate evidence-based preparedness and response capabilities to tackle risks from emerging and re-emerging pathogens and diseases, leading to improvement of the health of humans, animals, plants, and the environment at systems level | 0.98 | 0.93 | 0.91 | 0.92 | 0.91 | 0.93 |
| 120 | Develop a pathogen monitoring framework for wildlife and the environment, including in wildlife habitats and along farming and trade routes and the wild meat and products value chain and support the country in its implementation | 0.96 | 0.93 | 0.89 | 0.91 | 0.83 | 0.90 |
| 121 | Conduct target training for One Stop Border Post Port Health (vet and human) workforce to improve efficient work delivery | 0.96 | 0.92 | 0.88 | 0.92 | 0.88 | 0.92 |
| 122 | Support the development of core modules on environment, biodiversity and ecosystem health in the medical, veterinary and public health academic curricula and research agendas. | 1.00 | 0.92 | 0.98 | 0.96 | 0.92 | 0.96 |
| 123 | Develop a One Health needs assessment toolkit to evaluate interoperability, mechanisms and working relationships among sectors at country level | 1.00 | 0.92 | 0.93 | 0.94 | 0.91 | 0.94 |
| 124 | Support review of staffing needs at county level in accordance with functional and operational needs | 0.94 | 0.92 | 0.86 | 0.88 | 0.89 | 0.90 |
| 125 | Coordinate the national contribution to global One Health response to AMR | 0.94 | 0.92 | 0.87 | 0.87 | 0.88 | 0.90 |
| 126 | Conduct monitoring and evaluation and reporting of the Global Action Plan on AMR | 1.00 | 0.92 | 0.92 | 0.89 | 0.81 | 0.91 |
| 127 | Support shipment of samples for confirmation to regional support laboratories | 0.94 | 0.92 | 0.81 | 0.85 | 0.84 | 0.87 |
| 128 | Utilize global food safety campaign to sensitize and educate about the use of the One Health approach in this area among different stakeholders | 0.94 | 0.92 | 0.91 | 0.89 | 0.90 | 0.91 |
| 129 | Develop detailed annual investment and action plans with detailed justification for prioritised areas of investment, human resources and operational costs at each division and level within the Vet Service | 0.96 | 0.91 | 0.91 | 0.89 | 0.81 | 0.90 |
| 130 | Support the development of legal, sustainable, resilient and inclusive, wildlife-based economies while managing the risks of unregulated and illegal wildlife farming and trade | 0.97 | 0.91 | 0.89 | 0.82 | 0.89 | 0.89 |
| 131 | Map interoperability between health, animal disease and environment databases and information systems | 0.96 | 0.91 | 1.00 | 0.88 | 0.91 | 0.93 |
| 132 | Promote adoption of climate-smart and environmentally sound health systems | 0.92 | 0.91 | 0.93 | 0.85 | 0.93 | 0.91 |
| 133 | Jointly promote the importance of enhancing the integrity of all ecosystems and the services they provide to support healthy and resilient populations of all species | 0.92 | 0.91 | 0.93 | 0.86 | 0.85 | 0.89 |
| 134 | Conduct case study on the contribution of LIMS-SILAB to disease detection and response mechanisms in Kenya (targeting CVL & 4 RVILs) | 1.00 | 0.91 | 0.84 | 0.93 | 0.86 | 0.91 |
| 135 | Support countries to implement enabling, evidence-based and gender-sensitive regulatory frameworks for the prevention and control of zoonotic epidemics/pandemics along the value chains, including livestock and wildlife | 0.97 | 0.91 | 0.89 | 0.90 | 0.92 | 0.92 |
| 136 | Provide guidance for an adequate use of the integrated One Health information, surveillance and emergency response systems considering humans, animals, food, plants and ecosystems | 0.93 | 0.91 | 0.89 | 0.91 | 0.83 | 0.89 |
| 137 | Support countries to explore new communication channels to emphasize the central role of food safety across the entire food system, in operational and governance decision-making at national and other levels, and to guide adequate food safety investments | 0.94 | 0.91 | 0.93 | 0.89 | 0.87 | 0.91 |
| 138 | Develop methodologies and tools to advocate for and promote political prioritization of One Health work in regional, national, and local sustainable development strategies and plans | 0.97 | 0.91 | 0.85 | 0.91 | 0.86 | 0.90 |
| 139 | Map the evidence on the socio-economic impacts of environmental degradation (including land use change, biodiversity loss, pollution and waste) and climate change) | 1.00 | 0.91 | 0.95 | 0.93 | 0.89 | 0.93 |
| 140 | Support establishment of 2 CASICs and facilitate operationalization/ work plan/ action plan development for the CASICs (Trans Nzoia and Uasin Gishu) | 0.99 | 0.91 | 0.91 | 0.91 | 0.77 | 0.90 |
| 141 | Engage with citizen science in data collection for monitoring the health of the environment to inform action | 0.94 | 0.91 | 0.94 | 0.79 | 0.94 | 0.90 |
| 142 | Facilitate information sharing through support of hosting and maintenance of the KAHN website. | 0.94 | 0.91 | 0.91 | 0.85 | 0.85 | 0.89 |
| 143 | Develop guidance on economic analyses to quantify the costs and benefits of preventive interventions and use the results to advocate for sustainable financing in these interventions | 1.00 | 0.91 | 0.94 | 0.92 | 0.88 | 0.93 |
| 144 | Develop an over-arching National One Health framework that links OH thematic areas (to include AMR and Food safety) to the One Health Strategic Plan for coherence | 0.97 | 0.91 | 0.96 | 0.92 | 0.89 | 0.93 |
| 145 | Faciliate participation of 3 DVS and FAO in cross border animal disease surveillance and control programmes among IGAD and EAC countries.Virtual attendance will be supported as directed by the secretariat | 0.96 | 0.90 | 0.89 | 0.83 | 0.84 | 0.88 |
| 146 | Support platform for biannual/annual joint consultation of national & subnational One Health team | 0.96 | 0.90 | 0.90 | 0.93 | 0.83 | 0.90 |
| 147 | Develop guidance on progressive control and management pathways that apply a One Health approach for existing and potentially re-emerging zoonotic diseases (e.g., zoonotic influenza viruses, MERS-CoV, SARS-CoV-2, Ebola, RVF, etc.) and support countries to implement | 0.95 | 0.90 | 0.85 | 0.84 | 0.76 | 0.86 |
| 148 | Support samples and data collection (target high production counties and counties at boarders points) (Busia, Narok, Kajiado, Migori, Taita Taveta Lamu, Tana River, Isiolo, Wajir, Marsabit, Turkana) | 0.95 | 0.90 | 0.77 | 0.82 | 0.84 | 0.86 |
| 149 | Develop and maintain country capacity related to managing biohazards, including the safe storage and transport of infectious substances according to applicable international standards and regulations | 1.00 | 0.90 | 0.83 | 0.93 | 0.82 | 0.90 |
| 150 | Support global advocacy efforts | 0.95 | 0.90 | 0.84 | 0.89 | 0.77 | 0.87 |
| 151 | Develop advocacy training and tools for environment decision-makers professionals to influence decision-makers in other sectors | 0.96 | 0.90 | 0.93 | 0.88 | 0.93 | 0.92 |
| 152 | Strengthen CDVSs and RVILs with some training, tools, consumables & resources capabilities to perform outbreak investigations, follow-up actions and laboratory confirmation of suspected notifiable diseases events | 0.94 | 0.90 | 0.84 | 0.75 | 0.83 | 0.85 |
| 153 | Support participation of 2 DVS staff from Molecular section at the CVL, virology department in bioinformatics training at Institute of Primate Research (IPR) | 0.98 | 0.90 | 0.87 | 0.85 | 0.80 | 0.88 |
| 154 | Support countries to build science-policy interfaces to ensure scientific knowledge, including from assessments, syntheses and reviews, are translated into action | 0.92 | 0.90 | 0.88 | 0.87 | 0.79 | 0.87 |
| 155 | Provide training of CVL and sub-national labs using WHO's Global Laboratory Leadership Program (GLLP) | 0.98 | 0.90 | 0.85 | 0.91 | 0.82 | 0.89 |
| 156 | Develop an interoperable One Health training course (FTP-WEBE) for in-service professionals (the complement to FETP, FETPV and FELTP) targeting professionals in Ministries responsible for natural resource management (wildlife, biodiversity, ecosystems, environment), climate and other environmental issues | 0.96 | 0.89 | 0.92 | 0.92 | 0.86 | 0.91 |
| 157 | Promote the national-level recognition of the human right to a clean, healthy and sustainable environment (as unanimously approved by the UN Human Rights Council in October 2021) | 0.95 | 0.89 | 0.95 | 0.83 | 0.96 | 0.92 |
| 158 | Conduct weekly analysis of surveillance data including the laboratory, KABS, AMR, and outbreak investigation and response | 0.93 | 0.89 | 0.93 | 0.92 | 0.86 | 0.91 |
| 159 | Support VMD to enforce statutory limitations on the distribution, retail sale and use of prescription only medicines (POM’s) to/by livestock keepers and unsupervised veterinary paraprofessionals and Community Disease Reporters (CDR). | 0.90 | 0.89 | 0.81 | 0.88 | 0.71 | 0.84 |
| 160 | Support review and revision of the VSVPA and regulations to provide for mandatory CE requirement for annual retention on the veterinary register. | 0.85 | 0.89 | 0.87 | 0.92 | 0.80 | 0.87 |
| 161 | Support biannual meetings to discuss performance of surveillance systems (National epidemiologist, sub-national disease reporters, Washington State University, CDC) | 0.96 | 0.89 | 0.90 | 0.89 | 0.76 | 0.88 |
| 162 | Provide resources and support to countries to link and integrate single sector and specialized disease programmes and health information systems | 0.88 | 0.89 | 0.93 | 0.87 | 0.86 | 0.89 |
| 163 | Support training and capacitation of the Policy Unit/Department in the Ministry of Agric & Livestock | 0.93 | 0.89 | 0.88 | 0.84 | 0.83 | 0.87 |
| 164 | Support countries to conduct a baseline assessment of their food control system, particularly on critical elements that affect human, animal, plant, and environmental health | 0.98 | 0.89 | 0.95 | 0.94 | 0.85 | 0.92 |
| 165 | Promote One Health task forces and working groups with clear mandate for internal coordination | 0.94 | 0.89 | 0.96 | 0.98 | 0.85 | 0.93 |
| 166 | Support e-learning for laboratory personnel through VLC on QA as informed by regional QA programme and national QA road map; LMT and ATLASS trainings | 0.94 | 0.89 | 0.89 | 0.93 | 0.81 | 0.89 |
| 167 | Support VMD to develop pathways to recruit and train sufficient number of Veterinary Drug Inspectors to improve the level of enforcement of existing regulations limiting the sale and use of POMs | 0.90 | 0.89 | 0.84 | 0.84 | 0.77 | 0.85 |
| 168 | Develop the animal health sector workforce strategy | 0.96 | 0.88 | 0.90 | 0.90 | 0.87 | 0.91 |
| 169 | Procure Portable field PCR for 1 sub-national veterinary laboratories (RVIL Kericho) | 1.00 | 0.88 | 0.81 | 0.84 | 0.79 | 0.86 |
| 170 | Establish partnerships with universities and research centres to fill knowledge gaps and monitor environmental impacts on health (both positive and negative) | 0.96 | 0.88 | 0.96 | 0.92 | 0.88 | 0.92 |
| 171 | Leverage the use and implementation of already existing capacity evaluation tools and roadmaps5 at all levels to accelerate control of endemic zoonotic, neglected tropical and vector-borne diseases | 0.99 | 0.88 | 0.84 | 0.90 | 0.83 | 0.89 |
| 172 | Conduct county level policy consultation meetings with OH stakeholders (Garrisa, Nyeri) | 0.98 | 0.88 | 0.90 | 0.80 | 0.83 | 0.88 |
| 173 | Support Kenya to conduct analysis of the legislation relevant for each sector, to identify potential gaps and issues that would need to be addressed to reduce the emergence and spillover of diseases | 0.93 | 0.88 | 0.85 | 0.82 | 0.80 | 0.89 |
| 174 | Communicate to decision makers at all levels, the importance and economic value of a healthy environment to promote healthy and resilient societies and economies | 0.90 | 0.88 | 0.88 | 0.90 | 0.87 | 0.89 |
| 175 | Undertake M&E of ongoing ISAVET trainings (during class, field and individual field projects) | 0.95 | 0.88 | 0.88 | 0.89 | 0.83 | 0.88 |
| 176 | Support dissemination of the veterinary laboratory policy and strategic plan | 0.98 | 0.88 | 0.84 | 0.88 | 0.80 | 0.88 |
| 177 | workforce mapping/ profiling exercise | 0.94 | 0.88 | 0.85 | 0.87 | 0.85 | 0.88 |
| 178 | Support maintanace/customization of LIMS with new user requirments (at CVL, all 6 RVILs) (HQ Budget) | 1.00 | 0.87 | 0.84 | 0.86 | 0.81 | 0.88 |
| 179 | Support investigation of further deployment of Community Disease Reporters (CDR) in the ASAL areas to compliment the shortage of qualified veterinary paraprofessionals in these areas | 0.85 | 0.87 | 0.81 | 0.87 | 0.81 | 0.84 |
| 180 | Conduct 2 post training workshops (This will be conducted after each ISAVET training which will be conducted twice a year) | 0.96 | 0.87 | 0.87 | 0.88 | 0.80 | 0.87 |
| 181 | Support the development and adoption of policies and legislation to protect the rights of Indigenous Peoples and local communities to sustainably use and trade in natural resources | 0.90 | 0.87 | 0.91 | 0.87 | 0.94 | 0.90 |
| 182 | Conduct 4 ISAVET NIPSC meetings (2 meetings will be physical in Nairobi and 2 will be virtual) | 0.97 | 0.87 | 0.83 | 0.90 | 0.77 | 0.87 |
| 183 | Develop standardized protocols and standard operating procedures for harmonized One Health research and data collection, to facilitate data generation, sharing, comparison and meta-analyses | 0.90 | 0.87 | 0.83 | 0.84 | 0.75 | 0.84 |
| 184 | Develop operational tools to support science based One Health coordinated strategic technical actions | 0.96 | 0.87 | 0.92 | 0.88 | 0.83 | 0.89 |
| 185 | Provide scientific and technical assistance with the aim to enhance the participation of countries in the standard-setting work of the Codex Alimentarius Commission and relevant work of the OIE and facilitate its implementation through a multisectoral coordinated approach | 0.96 | 0.86 | 0.90 | 0.92 | 0.86 | 0.90 |
| 186 | Develop and implement comprehensive annual communication plans between DVS and County veterinary authorities to ensure effective stakeholders information dissemination on important events and programmes for stakeholders to become more involved with developing animal health, veterinary public health and programmes | 0.92 | 0.86 | 0.86 | 0.89 | 0.85 | 0.88 |
| 187 | Explore opportunity for the veterinary authority to get emergency funding from the Ministry of Disaster Management | 0.86 | 0.86 | 0.84 | 0.85 | 0.84 | 0.85 |
| 188 | Support VMD to conduct product information reviews | 0.98 | 0.86 | 0.84 | 0.89 | 0.82 | 0.88 |
| 189 | Support establishment of mechanism within VSVPA and regulations to define direct and indirect supervision of veterinary paraprofessionals by registered public & private veterinarians | 0.90 | 0.85 | 0.78 | 0.91 | 0.79 | 0.85 |
| 190 | Ensure that systems thinking is a core module for academic and in-service One Health professionals | 0.95 | 0.84 | 0.93 | 0.84 | 0.83 | 0.88 |
| 191 | Conduct data analysis and report writing | 0.96 | 0.84 | 0.81 | 0.89 | 0.77 | 0.85 |
| 192 | Support review of ratio of managerial component of staffing relative to deployment in sub-Counties and wards in accordance with the needs at County level | 0.90 | 0.84 | 0.86 | 0.84 | 0.86 | 0.86 |
| 193 | Conduct M&E for the 1st cohort of ISAVET trainings | 0.90 | 0.83 | 0.77 | 0.82 | 0.76 | 0.82 |
